# Spontaneous Tumour Lysis Syndrome in a Multiple Myeloma

**DOI:** 10.1155/2016/9620520

**Published:** 2016-11-10

**Authors:** Can Huzmeli, Eylem Eliacik, Mustafa Saglam, Baris Doner, Ferhan Candan

**Affiliations:** ^1^Necip Fazıl City Hospital, Department of Nephrology, Kahramanmaras, Turkey; ^2^Necip Fazıl City Hospital, Department of Hematology, Kahramanmaras, Turkey; ^3^Department of Nephrology, Cumhuriyet University, Sivas, Turkey

## Abstract

The tumor lysis syndrome (TLS) is a collection of metabolic abnormalities that occur in consequence of the release of intracellular contents following lysis of tumor cells. TLS occurs spontaneously or after chemotherapy. Spontaneous TLS is uncommon occurrence in multiple myeloma (MM). We define a case of a 70-year-old woman patient who was found to have MM with spontaneous TLS, following a compression fracture of the T-12 vertebrae. While serum uric acid and phosphorous levels were high, low calcium levels were identified. There were also acute kidney injury and metabolic acidosis. Upon the diagnosis of TLS, she was treated with hydration, allopurinol, sodium bicarbonate, and calcium gluconate. The improvement of her laboratory data was observed. We submitted this case in order to draw attention to the presentation of MM with spontaneous TLS.

## 1. Introduction

Tumor lysis syndrome (TLS) is a serious, life threatening complication of cancer chemotherapy that typically occurs in highly proliferative tumors. TLS is extremely rare among patients with multiple myeloma (MM), because of their low proliferation of the plasma cells. However, some risk factors for TLS in MM patients, such as hyperproliferative disease, immature plasma cell morphology, circulating plasmablasts, unfavorable cytogenetics, and increased lactate dehydrogenase have been identified [1, 2].

The TLS occurs when malign cells release their contents into the bloodstream, either spontaneously or after cytotoxic therapy. Characteristic laboratory findings include hyperkalemia, hypocalcemia, hyperphosphatemia, and hyperuricemia. These electrolyte disturbances cause cardiac arrhythmias, kidney failure, seizures, and death due to multiorgan insufficiency [3].

Spontaneous TLS in the absence of cytotoxic therapy has also been reported. Spontaneous TLS is uncommon oncological tumor emergence. Spontaneous TLS is extremely rare in literature and its occurrence in MM setting has been reported as one case. We describe a case of a 70-year-old woman who was found to have MM with spontaneous TLS.

## 2. Case Presentation

A 70-year-old woman was presented with loin pain of 2 months and unable to stand and walk for 2 weeks, with no comorbidities. In her physical examination, there were blood pressure of 130/80 mmHg, body temperature of 37.3°C, a pulse of 86 bpm, and grade 3 motor weakness of lower limbs. Biochemical profile of the patient was identified as glucose 98 mg/dL, blood urea nitrogen 198 mg/dL, serum creatinine 3,5 mg/dL, uric acid 17,4 mg/dL total protein 9,8 gr/dL, serum albumin 4,0 gr/dL, calcium 6,5 mg/dL, phosphate 8,7 mg/dL, potassium 5,1 mmol/L, LDH 856 IU/L, white cell count 11 000 mm^3^/uL, Hb 5,7 gr/dL, Htc 17%, and platelet count 28 000/mm^3^. Arterial blood gas analysis was evaluated as metabolic acidosis (pH 7,22, HCO_3_ 13 mEq/L, PCO_2_ 27 mmHg, and PO_2_ 96 mmHg). A radiography of the thoracal spine showed a compression fracture of the T12 vertebra. In serum protein electrophoresis, M-band was documented. Serum immunoglobulin Ig G was 5,6 g/dL. Serum and urine protein immunofixation revealed kappa light chain. Bone marrow aspiration smear showed abnormal plasma cells. Bone marrow biopsy showed plasma cells accounting for 90% of all cells; normal erythroid and myeloid cells were markedly suppressed. Immunohistochemistry examinations revealed kappa positive.

Our patient fulfilled clinical (acute renal injury) and laboratory criteria (hyperuricemia, hyperphosphatemia, and hypocalcemia) of TLS according to the Cairo and Bishop criteria [4]. The diagnosis of MM and spontaneous TLS were established. Medical treatment was initiated upon of TLS. The patient was treated with hydration, allopurinol, calcium gluconate, and sodium bicarbonate. After one week of initiating treatment, laboratory data showed glucose 117 mg/dL, blood urea nitrogen 48 mg/dL, serum creatinine 0,9 mg/dL, uric acid 3,2 mg/dL, serum albumin 3,5 gr/dL, calcium 7,8 mg/dL, phosphate 2,7 mg/dL, potassium 4,9 mmol/L, LDH 724 IU/L, white cell count 5300 mm^3^/uL, Hb 10,4 gr/dL, Htc 30,3%, and platelet count 40 000/mm^3^. Arterial blood gas analysis was normal.

## 3. Discussion

TLS is most frequently associated with highly proliferative malignancies such as non-Hodgkin's lymphoma and acute lymphocytic leukemia. TLS is a life threatening rare complication of metabolic disturbances occurring in about 5–20% of all malignancies [5]. The most commonly used diagnostic laboratory (two or more) and clinical (one or more) criteria for TLS are those proposed by Cairo-Bishop (Table 1) [4]. Characteristic laboratory signs contain hyperuricemia, hyperkalemia, hyperphosphatemia, hypocalcemia, metabolic acidosis, and elevated lactate dehydrogenase. Clinically, patients can present with ordinary symptoms such as nausea, weakness, vomiting, and muscle cramps or life threatening cardiac arrhythmias, kidney insufficiency, seizures, and sudden death.

TLS is an oncologic emergency that is caused by massive malign cell lysis with the release of large amounts of phosphate, potassium, uric acid, and nucleic acids into the systemic circulation. Degradation of nucleic acid leads to hyperuricemia. The evident increase in urinary uric acid excretion can have an outcome in the precipitation of uric acid in the kidney tubules. Hyperuricemia can also induce renal vasoconstriction, inflammation, and decreased renal blood flow, occurring in acute renal injury. Hyperphosphatemia with calcium phosphate accumulation in the renal tubules can also lead to acute renal damage.

Spontaneous TLS is about 15% of all cases of TLS. Spontaneous TLS has been described in solid tumors and hematological tumors [6, 7]. Saravu et al. reported a case having MM with spontaneous TLS, following a compression fracture of the L2 vertebrae. Hyperuricemia, hyperkalemia, hyperphosphatemia, normocalcemia, and acute renal injury in their patient were identified [8]. Our case was the second one spontaneous TLS having a compression fracture of T12 with history of loin pain for two months. TLS is very rare in MM as it is indolent malignancy. However, high tumor burden, immature morphology, high proliferative activity, and poor cytogenetics may be precipitating factors [9]. Our case had extensive bone marrow plasmacytosis (plasma cells > 90% of all nucleated cells) with presence of plasmablasts. Also, endogenous secretion of glucocorticoid with infection and fever might have been trigger factor as to cause increased tumor lysis in some patients [10, 11].

Prevention of TLS begins with recognition of risk factors and close laboratory and clinical monitoring. Additionally, nonsteroidal anti-inflammatory drugs, iodinated radiocontrast matter, and other potentially nephrotoxic therapeutic agents should be avoided to abrogate the risk of AKI from TLS. Vigorous hydration, close monitoring serum electrolytes (calcium, potassium, and phosphate levels), and control of uric acid levels with allopurinol (300 mg/day) or rasburicase (0.1–0.2 mg/kg intravenously for one dose) are cornerstone of the treatment of TLS. Administration of fluid is significant because it increases renal blood flow and glomerular filtration and reduces the urinary supersaturation of calcium, phosphate, and uric acid. Target urine output must be ≥2 mL/kg/h. Diuretics may be necessary if patients develop volume overload. Alkalinization makes physiologic sense, as increasing urine pH can increase the solubility of uric acid. But urinary alkalinization is not recommended in the management of TLS. Because at the same time the solubility of calcium phosphate or xanthine may decrease and increase calcium phosphate precipitation in renal tubules. However, the use of sodium bicarbonate should be restricted to those patients with severe metabolic acidosis. Allopurinol has been shown to prevent hyperuricemia due to cytotoxic chemotheraphy although it does not reduce preexisting hyperuricemia. Rasburicase is a recombinant uric acid oxidase which directly causes conversion of uric acid into allantoin, much more soluble substance. The effect of rasburicase starts within 4 h and uric acid normalized within 3-4 days. In patients with a prior history of glucose-6-phosphate dehydrogenase, rasburicase is contraindicated and allopurinol should be utilized instead of rasburicase [6, 12].

We did not give rasburicase to our patient because it is not sold commonly in the market of our country. Hemodialysis is needed in established cases of TLS with AKI complicated by severe oliguria, anuria, life-threatening hyperkalemia, or hyperphosphatemia-induced hypocalcemia, if there was no response to all medical drug treatments [1].

## 4. Conclusion

We report an adult presenting with AKI and acute TLS before her diagnosis of MM, in the absence of precipitating cytotoxic chemotheraphy or radiotherapy. It has been considered occult malignancy in the case of unexplainable AKI with hyperuricemia. Upon TLS suspicion, prompt recognition and aggressive treatment with intravenous hydration and hypouricemic agents and judicious hemodialysis treatment are critical for successful outcome.

## Figures and Tables

**Table 1:** 2004 Cairo-Bishop laboratory and clinical criteria for tumor lysis syndrome [4].

*Laboratory tumor lysis syndrome criterion for diagnosis*
Uric acid: ≥8 mg/dL or 25% increase from baseline
Potassium: ≥6 mEq/L or 25% increase from baseline
Phosphorus: ≥6,5 mg/dL (children), ≥4.5 mg/dL (adults), or 25% increase from baseline
Calcium: ≤7 mg/dL, ≤25% decrease from baseline
*Clinical tumor lysis syndrome criterion for diagnosis*
Creatinine ≥1.5 upper limit of normal (age > 12 years of age or age adjusted)
Cardiac arrhythmia or sudden death
Seizure
